# Prognosis after ST-elevation myocardial infarction: a study on cardiac magnetic resonance imaging versus clinical routine

**DOI:** 10.1186/1745-6215-15-249

**Published:** 2014-06-25

**Authors:** Suzanne de Waha, Ingo Eitel, Steffen Desch, Georg Fuernau, Philipp Lurz, Thomas Stiermaier, Stephan Blazek, Gerhard Schuler, Holger Thiele

**Affiliations:** 1 Department of Internal Medicine/Cardiology, University of Leipzig - Heart Center, Strümpellstr. 39, 04289 Leipzig, Germany; 2 Department of Cardiology and Angiology, Heart Center Bad Segeberg, Am Kurpark 1, 23795 Bad Segeberg, Germany; 3 Medical Clinic II, University of Lübeck, Ratzeburger Allee 160, 23538 Lübeck, Germany

**Keywords:** ST-elevation myocardial infarction, Prognosis, Traditional outcome markers, Cardiac magnetic resonance imaging

## Abstract

**Background:**

This study aimed to evaluate the incremental prognostic value of infarct size, microvascular obstruction (MO), myocardial salvage index (MSI), and left ventricular ejection fraction (LV-EF_CMR_) assessed by cardiac magnetic resonance imaging (CMR) in comparison to traditional outcome markers in patients with ST-elevation myocardial infarction (STEMI) reperfused by primary percutaneous intervention (PCI).

**Methods:**

STEMI patients reperfused by primary PCI (n = 278) within 12 hours after symptom onset underwent CMR three days after the index event (interquartile range [IQR] two to four). Infarct size and MO were measured 15 minutes after gadolinium injection. T2-weighted and contrast-enhanced CMR were used to calculate MSI. In addition, traditional outcome markers such as ST-segment resolution, pre- and post-PCI Thrombolysis In Myocardial Infarction (TIMI)-flow, maximum level of creatine kinase-MB, TIMI-risk score, and left ventricular ejection fraction assessed by echocardiography were determined in all patients. Clinical follow-up was conducted after 19 months (IQR 10 to 27). The primary endpoint was defined as a composite of death, myocardial reinfarction, and congestive heart failure (MACE).

**Results:**

In multivariable Cox regression analysis, adjusting for all traditional outcome parameters significantly associated with the primary endpoint in univariable analysis, MSI was identified as an independent predictor for the occurrence of MACE (Hazard ratio 0.94, 95% CI 0.92 to 0.96, *P* <0.001). Further, C-statistics comparing a model including only traditional outcome markers to a model including CMR parameters on top of traditional outcome markers revealed an incremental prognostic value of CMR parameters (0.74 versus 0.94, *P* <0.001).

**Conclusions:**

CMR parameters such as infarct size, MO, MSI, and LV-EF_CMR_ add incremental prognostic value above traditional outcome markers alone in acute reperfused STEMI.

**Trial registration:**

Clinicaltrials.gov NCT00463749, Clinicaltrials.gov NCT00359918.

## Background

ST-elevation myocardial infarction (STEMI) remains a common cause of death worldwide. Although mortality in the acute phase has declined in recent years, partly due to rapid reperfusion by primary percutaneous coronary intervention (PCI), longterm prognosis remains poor [1]. Early identification of patients at high risk for adverse clinical outcome ideally followed by intensification of therapeutic measures offers the potential to improve prognosis. In clinical routine, risk stratification is commonly performed using echocardiographic, electrocardiographic, laboratory, and angiographic parameters or established risk scores [2-5]. These outcome markers are relatively easy to obtain, requiring only moderate financial and human resources. Cardiac magnetic resonance imaging (CMR) offers a variety of markers such as infarct size, microvascular obstruction (MO), and myocardial salvage index (MSI). These CMR parameters have been shown to be robust predictors of adverse clinical outcome in mid-sized single center studies [6-9], but are not commonly incorporated in classic models of clinical risk assessment. Moreover, left-ventricular ejection fraction assessed by CMR (LV-EF_CMR_) might lead to more accurate results in comparison to assessment by echocardiography [10,11]. However, due to significant costs and requirements in term of infrastructure and personnel resources, acquisition of these CMR-derived markers in a purely clinical setting would only be justified if CMR parameters offered an additional prognostic value above traditional outcome parameters. If so, CMR might allow for an improved identification of patients at high risk for recurrent cardiovascular events, influencing further clinical management. However, the prognostic value of CMR-derived parameters in comparison to a detailed set of traditional non-CMR markers including left-ventricular ejection fraction determined by echocardiography (LV-EF_echo_), enzymatic infarct size, and the Thrombolysis In Myocardial Infarction (TIMI)-risk score assessed in clinical routine, has not yet been investigated. The aim of the current study was therefore to evaluate a potential incremental prognostic value of CMR parameters such as infarct size, MO, MSI, and LV-EF_CMR_ in comparison to traditional outcome markers in a large cohort of patients with STEMI reperfused by PCI.

## Methods

### Patients and design

Data from 512 consecutive STEMI patients undergoing primary PCI at our tertiary care center were analyzed. Inclusion criteria were the presence of symptoms for less than 12 hours and ST-segment elevation in more than or equal to two leads with more than or equal to 0.2 mV in precordial or more than or equal to 0.1 mV in extremity leads. Patients with prior fibrinolysis, prior myocardial infarction, and contraindications to CMR at study entry were excluded. The current retrospective analysis is based on patients that were in part initially enrolled in the LIPSIA N-ACC trial investigating the impact of high-dose N-acetylcysteine on MSI and the LIPSIA-STEMI trial investigating the impact of pre-hospital initiated facilitated PCI versus primary PCI on infarct size and MSI [12,13]. The LIPSIA N-ACC trial was negative as the intervention did not result in an improved reperfusion success. Further, patients of the LIPSIA-STEMI trial included in the current analysis were enrolled in the control arm and treated by standard STEMI therapy with primary PCI within 12 hours after symptom onset in accordance with current guidelines.

Prior to PCI, all patients received 500 mg of aspirin and activated clotting time-adjusted unfractionated heparin intravenously. The intake of 100 mg of aspirin indefinitely plus clopidogrel with a 600 mg loading-dose reducing to a daily dose of 75 mg for 12 months were mandatory. All other medication including glycoprotein IIb/IIIa-inhibitors were administered according to current guidelines [14]. Primary PCI was performed according to standard clinical practice. Additional thrombectomy was used depending on the thrombus burden in the infarct-related artery.

This study complies with the Declaration of Helsinki and was approved by the University of Leipzig ethics committee. All patients gave written informed consent.

### Assessment of traditional outcome markers

All patients underwent detailed assessment of medical history as well as physical examination at index hospitalization. Hypertension, hyperlipidemia, diabetes, and smoking status were assessed using previous definitions [15]. Serum creatinine was measured at admission using the Jaffe method (Roche Diagnostics, Mannheim, Germany). The TIMI-risk score was calculated for all patients as previously described [3]. Early ST-segment resolution, expressed as percentage change from pre to post PCI, was evaluated by measuring the sum of ST-segment elevation 20 ms after the end of the QRS complex in the electrocardiogram before and after PCI [5]. Creatine kinase-MB (CK-MB) was assessed using the standard photometric immunological UV-test (Roche Diagnostics, Mannheim, Germany). Enzymatic infarct size was defined as the maximum level of CK-MB (CK-MB_max_) derived from measurements every six hours over two days. Pre and post PCI coronary angiography of the target lesion was performed with the same projections allowing optimal offline evaluation of the TIMI-flow of the infarct-related artery by two independent observers [16].

For determination of LV-EF_echo_ all patients underwent transthoracic examinations 24 to 48 hours after the index event. An echocardiography was performed by independent experienced observers according to standard clinical practice in accordance with the American Society of Echocardiography standards using Vivid-7 ultrasound equipment (GE, Milwaukee, Illinois, United States) [17]. LV-EF_echo_ was assessed in standard apical two- and four-chamber views capturing at least three cardiac cycles by using the modified Simpson’s rule.

The time-to-revascularization was calculated in all patients and was defined as time from symptom onset or climax of symptoms in case of recurrent pre-infarction angina to first balloon inflation.

### Cardiac magnetic resonance imaging

All patients were examined at rest in the supine position with a whole body 1.5-Tesla MR scanner (Gyroscan Intera CV, Philips Medical Systems, Best, Netherlands) equipped with a five-element cardiac phased-array coil for signal reception. A vectorcardiogram for gating and triggering was used. To define the orientation of the heart, a realtime interactive tool was applied. All images were acquired during breath-hold at end exspiration.

Assessment of LV-EF_CMR_ was performed by a standard steady-state free precession technique acquiring short-axis slices from base to apex as well as horizontal and vertical long axis views. Delayed enhancement short-axis images covering the whole ventricle were acquired at the mid-diastole approximately 15 minutes after the bolus injection (0.2 mmol/kg/bodyweight) of gadobutrol (Gadovist, Schering, Leverkusen, Germany). An inversion-recovery (IR) turbo gradient echo sequence was used for image acquisition. The individual IR prepulse delay was defined in order to obtain the maximal contrast between viable and necrotic myocardium. Short-axis slices covering the whole left ventricle using a T2-weighted imaging triple inversion recovery turbo spin-echo sequence before contrast administration were obtained, allowing the assessment of edema or area-at-risk.

CMR measurements were performed offline in a core lab by operators blinded to the baseline and outcome data using a dedicated CMR evaluation software (View-Forum release 5.2, Philips Medical Systems, Washington, United States). A semi-automated computer-aided approach was used to identify the regions of edema and infarcted myocardium, as previously described [8,18]. Myocardial edema was defined as a mean signal intensity of more than 2 SD of remote myocardium in T2-weighted images. The myocardium was considered infarcted if the signal intensity was more than 5 SD above remote myocardium in delayed enhancement images. MO was assessed approximately 15 minutes after gadolinium injection, again using delayed enhancement images. In patients with MO, the areas of hypo-enhancement within the hyper-enhanced infarct region were included for infarct size analysis. Infarct size, area-at-risk, and MO were expressed as percentages of the left ventricular mass (%LV), given by the sum of the mass of edema, late enhancement, and MO regions for all slices divided by the sum of the LV myocardial cross-sectional mass. MSI was calculated as area-at-risk minus infarct size divided by area-at-risk as described previously [19]. LV-EF_CMR_ was calculated from the short-axes functional views. The performance of the CMR core lab has been demonstrated previously [20,21].

### Endpoints and definitions

Clinical follow-up was conducted via a structured questionnaire by telephone. Any clinical event was verified by hospital charts, direct contact with the treating physician, or contact with the local government registration. The follow-up interviewer was not aware of baseline or CMR data. All outcomes were adjudicated by a clinical events committee.

The primary endpoint was defined as any major adverse cardiovascular event (MACE) including death, non-fatal myocardial reinfarction, or congestive heart failure. The key secondary endpoint was death. Death was regarded as cardiac in origin unless obvious non-cardiac causes could be identified. The diagnosis of reinfarction during the index hospitalization was based on clinical symptoms, new ST-segment changes, and an increase in the CK-MB levels above the reference limits in patients with normalized values or if there was an increase of more than 20% from the last non-normalized measurement. At follow-up, any new ischemic symptoms leading to hospital admission accompanied by elevated troponin T was defined as a reinfarction [22]. New congestive heart failure was defined as congestive heart failure (= rales, dyspnea, New York Heart Association functional class III to IV) requiring medical attention and treatment with diuretics occurring more than 24 hours after the index event. In patients experiencing more than one event, the first event was chosen for the combined clinical endpoint. When one or more events occurred simultaneously, the most severe event was chosen (death, followed by reinfarction, followed by congestive heart failure).

### Statistical analysis

Each categorical variable is expressed as the number and percentage of patients. Continuous data are reported as medians with the corresponding interquartile range (IQR). Two-group comparisons were performed with Chi-square tests for categorical variables, Student *t* tests for normally distributed continuous variables and Wilcoxon rank-sum tests for non-normally distributed continuous variables. The correlation of enzymatic infarct size and infarct size assessed by CMR, as well as of LV-EF_echo_, and LV-EF_CMR_, were assessed by Spearman’s correlation coefficients.

Further, univariable and multivariable Cox regression analyses with stepwise inclusion were performed including all parameters as continuous variables to investigate the relation of traditional outcome markers and CMR parameters with the time-dependent occurrence of MACE and mortality. All variables with a *P* value <0.05 in univariable analysis entered the multivariable model. Finally, C-statistics according to DeLong [23] were performed to analyze the prognostic value in predicting MACE and mortality of a model including only traditional outcome parameters (CK-MB_max_, TIMI-risk score, ST-segment resolution, TIMI-flow pre-PCI, TIMI-flow post-PCI, LV-EF_echo_) when compared with a second model including CMR parameters (LV-EF_CMR_, infarct size, MO, and MSI) on top of the first model [23].

All statistical tests were performed with SPSS software, version 17.0 (SPSS Inc., Chicago, Illinois, United States) and MedCalc software, version 12.2.1 (MedCalc Software, Ostend, Belgium). All probability values were two-tailed with α = 0.05 and all confidence intervals (CI) were calculated to the 95th percentile.

## Results

Of 512 eligible consecutive patients undergoing primary PCI for STEMI, CMR was conducted in 438 patients. The reasons for a lack of CMR were claustrophobia (n = 19), death prior to CMR (n = 18), refusal (n = 15), pacemaker (n = 5), obesity (n = 7) and reasons that could not be further clarified (n = 10). Due to a prior myocardial infarction, 29 additional patients were excluded. T2-weighted imaging covering the whole left ventricle was performed in 287 patients. T2-weighted images of 29 patients were of poor quality, but judged to be analyzable. Finally, follow-up was completed in 278 (97%) patients.

### Baseline characteristics

Baseline characteristics are displayed in Table 1. CMR was performed in median three days after the index event (IQR two to four). Within the follow-up period of 19 months (IQR 10 to 24), 52 events occurred (death n = 18, reinfarction n = 17, and new congestive heart failure n = 17 patients). LV-EF_echo_ and LV-EF_CMR_, as well as enzymatic infarct size and infarct size assessed by CMR, were shown to be only moderately correlated (r = 0.63, *P* <0.001 and r = 0.69, *P* <0.001 respectively).

**Table 1:** Baseline characteristics and CMR parameters of the whole study cohort and according to the occurrence of MACE

**Variable**	**All patients (n = 278)**	**MACE + (n = 52)**	**MACE – (n = 226)**	***P *** **value**
Age, years	65 (55–73)	69 (60–77)	64 (55–72)	0.02
Male sex, n (%)	201 (72.3)	36 (69.2)	164 (72.9)	0.27
Hypertension, n (%)	190 (68.6)	39 (75.0)	224 (67.0)	0.29
Diabetes mellitus, n (%)	74 (26.7)	21 (40.4)	53 (23.7)	0.04
Hyperlipidemia, n (%)	88 (31.8)	21 (40.4)	66 (29.5)	0.20
Current smoking, n (%)	104 (37.5)	14 (26.9)	89 (39.7)	0.17
Serum creatinine, μmol/l	80 (68–91)	83 (69–99)	79 (68–90)	0.18
BMI, kg/m^2^	27 (25–30)	27 (26–30)	27 (25–30)	0.80
CK-MB_max_, μmol/l	3.0 (1.4–5.2)	4.5 (1.6–6.7)	2.8 (1.6–5.0)	<0.001
TIMI-risk score	3.0 (2.0–5.0)	5.0 (3.0–7.0)	3.0 (2.0–5.0)	<0.001
ST-segment resolution, %	73 (50–100)	62 (32–77)	76 (50–100)	0.003
Angiographic findings, n (%)				
TIMI-flow pre-PCI 0	147 (52.8)	33 (63.4)	115 (50.9)	0.03
TIMI-flow pre-PCI I-III	131 (47.1)	19 (36.5)	111 (49.1)	
TIMI-flow post-PCI 0-II	36 (12.9)	39 (75.0)	23 (10.6)	0.002
TIMI-flow post-PCI III	242 (87.0)	13 (25.0)	203 (89.8)	
LV-EF_echo_, %	45 (40–50)	40 (32–50)	46 (40–55)	0.001
Time-to-revascularization, min	193 (132–348)	211 (135–396)	190 (128–336)	0.35
CMR parameters				
LV-EF_CMR_, %	52 (42–60)	41 (33–50)	54 (45–62)	<0.001
Infarct size, %LV	16.1 (8.2–26.6)	31.5 (22.5–40.0)	13.1 (5.7–22.4)	<0.001
MO, %LV	0.69 (0.1–1.6)	1.5 (0.6–2.9)	0.6 (0.0–1.5)	0.001
MSI	48.0 (27.3–73.5)	27.0 (10.0–;39.7)	67.4 (47.9–83.3)	<0.001

Continuous data are presented as median and interquartile range. %LV = percentage of left ventricular mass; BMI = body mass index; CK-MB_max_ = maximum level of creatine kinase-MB; CMR = cardiac magnetic resonance imaging; LV-EF_CMR_ = left ventricular ejection fraction assessed by CMR; LV-EF_echo_ = left ventricular ejection fraction assessed by echocardiography; MACE = major adverse cardiac events; MO = microvascular obstruction; MSI = myocardial salvage index; PCI = percutaneous coronary intervention; TIMI = Thrombolysis In Myocardial Infarction. Traditional outcome markers and prognosis

Patients in whom the primary composite endpoint occurred displayed significantly lower values of ST-segment resolution, higher levels of CK-MB_max_, higher prevalence of TIMI-flow pre-PCI 0, and lower prevalence of TIMI-flow post-PCI III in comparison to patients remaining event-free (Table 1). Finally, the TIMI-risk score was significantly higher in patients who experienced an event, whereas LV-EF_echo_ was significantly lower.

The results of the univariable Cox regression analyses on the association of traditional outcome markers with the occurrence of MACE and mortality are displayed in Tables 2 and 3.

**Table 2:** Traditional outcome markers and CMR parameters: association with MACE in univariable and stepwise multivariable Cox regression analysis

	**Univariable analysis**		**Multivariable analysis**	
**Variable**	**HR (95% CI)**	***P *** **value**	**HR (95% CI)**	***P *** **value**
Male gender	0.97 (0.53–1.77)	0.97	not included	-
Hyperlipidemia	1.42 (0.82–2.47)	0.21	not included	-
Serum creatinine, μmol/l	1.01 (0.99–1.02)	0.23	not included	-
CK-MB_max_, μmol/l	1.02 (1.01–1.03)	0.01	-	-
TIMI-risk score	1.27 (1.15–1.41)	<0.001	-	-
ST-segment resolution, %	0.98 (0.97–0.99)	0.001	-	-
TIMI-flow pre-PCI 0	1.24 (1.01–1.53)	0.04	-	-
TIMI-flow post-PCI 0-II	2.49 (1.44–4.31)	0.001	-	-
LV-EF_echo_, %	0.95 (0.92–0.97)	<0.001	-	-
Time-to-revascularization, min	1.00 (0.99–1.01)	0.44	not included	-
LV-EF_CMR_, %	0.96 (0.94–0.97)	<0.001	-	-
Infarct size, %LV	1.07 (1.05–1.09)	<0.001	-	-
MO, %LV	1.16 (1.07–1.25)	<0.001	-	-
MSI	0.93 (0.92–0.95)	<0.001	0.94 (0.92–0.96)	<0.001

%LV = percentage of left ventricular mass; CK-MB_max_ = maximum level of creatine kinase-MB; CI = confidence interval; CMR = cardiac magnetic resonance imaging; HR = hazard ratio; LV-EF_CMR_ = left ventricular ejection fraction assessed by CMR; LV-EF_echo_ = left ventricular ejection fraction assessed by echocardiography; MACE = major adverse cardiac events; MO = microvascular obstruction; MSI = myocardial salvage index; PCI = percutaneous coronary intervention; TIMI = Thrombolysis In Myocardial Infarction.

**Table 3:** Traditional outcome markers and CMR parameters: association with mortality in univariable and stepwise multivariable Cox regression analysis

	**Univariable analysis**		**Stepwise multivariable analysis**	
**Variable**	**HR (95% CI)**	***P *** **value**	**HR (95% CI)**	***P *** **value**
Male gender	1.24 (0.42–3.60)	0.70	not included	-
Hyperlipidemia	0.81 (0.29–2.27)	0.69	not included	-
Serum creatinine, μmol/l	1.01 (0.98–1.03)	0.64	not included	-
CK-MB_max_, μmol/l	1.04 (0.99–1.08)	0.08	-	-
TIMI-risk score	1.31 (1.07–1.60)	0.009	-	-
ST-segment resolution, %	0.99 (0.98–1.01)	0.07	not included	-
TIMI-flow pre-PCI 0	1.20 (0.83–1.75)	0.32	not included	-
TIMI-flow post-PCI 0-II	4.39 (1.73–11.15)	0.002	-	-
LV-EF_echo_, %	0.94 (0.89–0.98)	0.005	-	-
Time-to-revascularization, min	1.00 (0.99–1.01)	0.25	not included	-
LV-EF_CMR_, %	0.95 (0.92–0.98)	0.003	-	-
Infarct size, %LV	1.07 (1.04–1.10)	<0.001	-	-
MO, %LV	1.09 (1.02–1.21)	0.01	-	-
MSI	0.93 (0.92–0.95)	<0.001	0.93 (0.90–0.95)	<0.001

%LV = percentage of left ventricular mass; CI = confidence interval; CK-MB_max_ = maximum level of creatine kinase-MB; CMR = cardiac magnetic resonance imaging; HR = hazard ratio; LV-EF_CMR_ = left ventricular ejection fraction assessed by CMR; LV-EF_echo_ = left ventricular ejection fraction assessed by echocardiography; MO = microvascular obstruction; MSI = myocardial salvage index; PCI = percutaneous coronary intervention; TIMI = Thrombolysis In Myocardial Infarction.

### CMR parameters and prognosis

Patients with MACE had a significantly larger infarct size, higher extent of MO, and lower values of MSI, as well as a lower LV-EF_CMR_ (Table 1). LV-EF_CMR_, infarct size, MO, and MSI were significantly associated with the time-dependent occurrence of MACE and mortality in univariable analysis (Tables 2 and 3).

**Figure 1:**
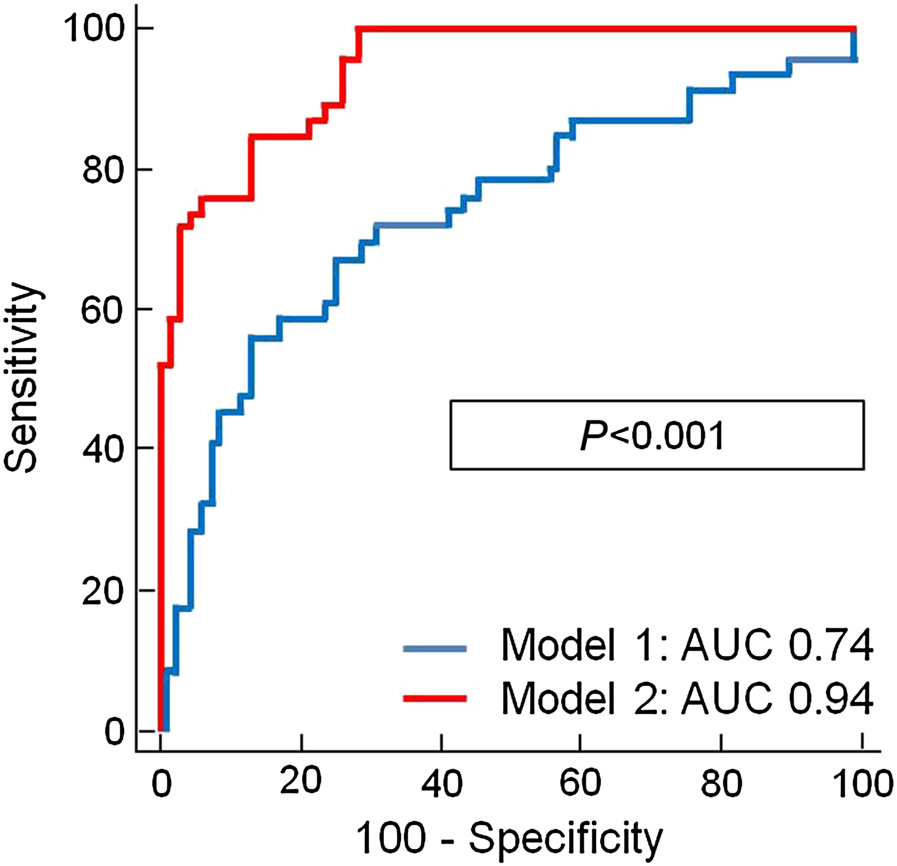
**C-statistics comparing the prognostic value for MACE prediction of model 1 (CK-MB**_**max**_**, TIMI-risk score, ST-segment resolution, TIMI-flow pre-PCI, TIMI-flow post-PCI, LV-EF**_**echo**_**) and model 2 (model 1 + LV-EF**_**CMR**_**, infarct size, MO and MSI).**

**Figure 2:**
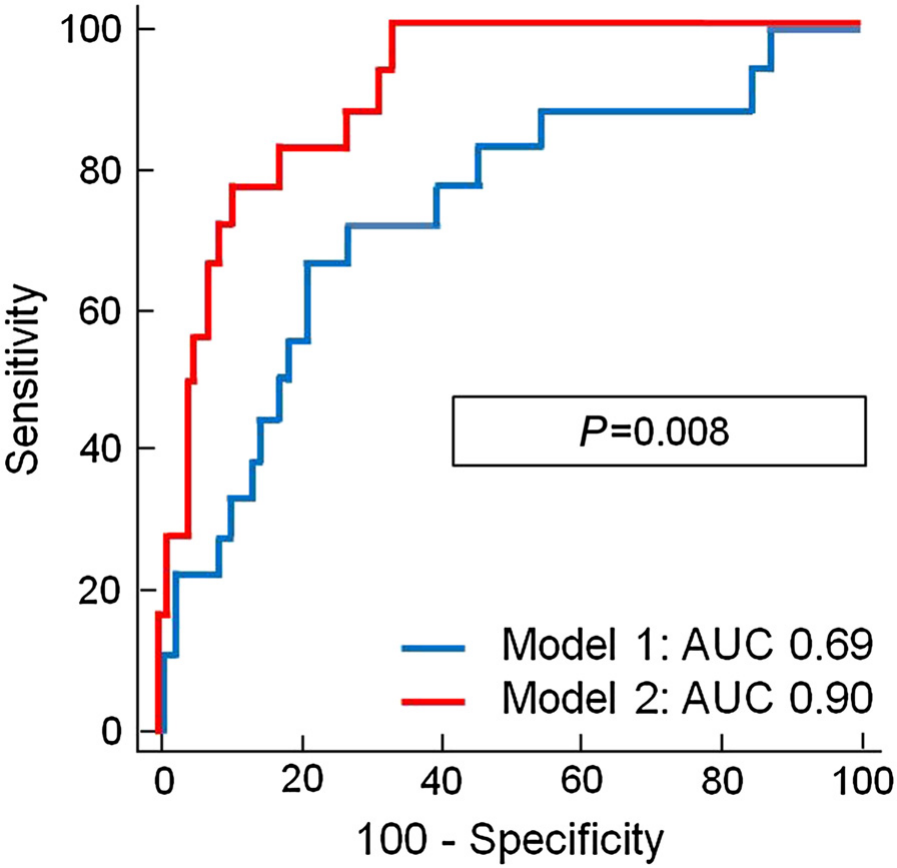
**C-statistics comparing the prognostic value for mortality prediction of model 1 (CK-MB**_**max**_**, TIMI-risk score, ST-segment resolution, TIMI-flow pre-PCI, TIMI-flow post-PCI, LV-EF**_**echo**_**) and model 2 (model 1 + LV-EF**_**CMR**_**, infarct size, MO and MSI).**

In stepwise multivariable Cox regression analyses, MSI was identified as an independent predictor for the time-dependent occurrence of MACE and mortality (Tables 2 and 3). C-statistics analyzing the prognostic value to predict MACE and mortality of a model including only traditional outcome markers (model 1: CK-MB_max_, TIMI-risk score, ST-segment resolution, TIMI-flow pre-PCI, TIMI-flow post-PCI, and LV-EF_echo_) to a model including CMR parameters on top of the traditional outcome markers (model 2: model 1 + LV-EF_CMR_, infarct size, MO, and MSI) demonstrated an incremental prognostic value of CMR parameters above traditional outcome markers (MACE: 0.74 versus 0.94, *P* <0.001; mortality: 0.69 versus 0.90, *P* = 0.008, Figures 1 and 2).

## Discussion

The main findings of this study can be summarized as follows: 1) infarct size, MO and MSI as well as LV-EF_CMR_ are associated with the time-dependent occurrence of death, reinfarction and congestive heart failure after STEMI; 2) MSI is a strong independent predictor for MACE and mortality; and 3) CMR parameters such as infarct size, MO, MSI, and LV-EF_CMR_ add incremental prognostic value above traditional outcome markers only.

Although in recent years substantial progress with regard to short-term outcome has been made in patients with STEMI, longterm prognosis has not been significantly altered and remains unsatisfactory despite the implementation of new primary and secondary prevention strategies [1]. Thus, increasing emphasis has been put on optimized risk stratification, as this offers the potential to further improve outcome by identifying patients who are at high risk. In clinical routine this is mainly performed by electrocardiographic and echocardiographic tests, as well as laboratory and angiographic parameters or established risk scores [2-5]. In contrast to these indirect markers of reperfusion success, CMR offers the possibility to directly visualize and quantify infarct expansion and microvascular impairment, as well as the amount of salvaged myocardium. Previous mid-sized single center studies have shown that all of these CMR parameters of myocardial damage are associated with adverse functional and clinical outcome [6-9]. CMR parameters might therefore present a powerful tool for detecting patients at the highest risk of a recurrent cardiovascular event. To date, there are few data on the potential incremental value of CMR parameters above traditional outcome markers. Given the high cost of CMR, evidence of such additional prognostic value would be a prerequisite for a more widespread use of CMR in the assessment of prognosis outside the research setting. However, previous studies analyzing the prognostic value of CMR parameters are strongly limited due to the incomplete inclusion of traditional outcome markers [6-9]. Further, these studies led to inconsistent results. In detail, Larose *et al.* identified infarct size as the best predictor for adverse outcome in comparison to traditional prognostic markers [9]. However, parameters such as established clinical risk scores, ST-segment resolution or post-procedural angiographic results were not included. When interpreting these data it is important to mention that MSI was assessed by acquiring only three slices of T2-weighted images to identify the area-at-risk and/or edema, which might have significantly influenced the results [9]. In addition, the combined endpoint included relatively weak components such as LV-EF of less than 35% at follow-up. Finally, the study cohort comprised of only 103 patients. In contrast, Masci *et al.* could identify MSI as an independent predictor for adverse functional outcome and ST-segment resolution [24]. In line with these results, Eitel *et al.* demonstrated a clear independent association of MSI with adverse clinical outcome in 208 patients [7,8]. However, in these studies, adjustment for a broad spectrum of traditional outcome markers was not performed. The assessment of LV-EF and infarct size can be conducted by alternative methods such as transthoracic echocardiography and laboratory analysis of cardiac enzymes. Both parameters are known to have a high prognostic impact and represent clinical standard, requiring only moderate financial and personnel resources in comparison to the rather complex assessment of infarct size and LV-EF by CMR. Besides LV-EF_echo_ and enzymatic infarct size, the TIMI-risk score is also known to be a strong predictor for clinical outcome and its assessment can be easily performed within the daily clinical routine requiring virtually no additional expense. Nevertheless, the prognostic value of CMR parameters including MSI has so far never been compared to this well-validated clinical risk score. Thus, the current study expands on previous findings as we could demonstrate that MSI is a strong independent predictor for MACE and mortality even after adjustment for a detailed and comprehensive set of traditional prognostic parameters.

Moreover, the current study is the first to demonstrate that CMR parameters offer incremental prognostic value for MACE and mortality in addition to the methods for risk stratification commonly performed in clinical routine. Although infarct size, MO, or MSI have not directly been linked to differing treatment strategies, they offer the potential to identify patients requiring more intensive medical care in the subacute and chronic phase after STEMI. Finally, in the light of the prognostic value, the current results underline the role of CMR parameters as surrogate endpoints for clinical trials apart from further risk stratification in clinical patient-based routine.

Some limitations of the current trial need to be addressed. First, these data arise from a single-center cohort and the sample size is still too small to reach definitive conclusions; however, it is the largest cohort reported so far. Second, CMR data were acquired during the first days after STEMI with some heterogeneity between the individual examinations. Although data on the natural evolution of infarct size, MO, and MSI after STEMI are scarce and ambiguous, performing CMR at a singular exact time point after STEMI might lead to the most accurate results. Third, CMR image analysis was performed using a semi-automated approach. Alternative methods exist including the full width at half maximum technique or manual delineation. However, the optimal technique for CMR image analysis has not yet been established [25]. In addition, the threshold for the determination of infarcted myocardium used in the current analysis is in line with the recommendation of the Society of Cardiovascular Magnetic Resonance for semi-automated analysis in myocardial infarction [25]. Moreover, as the different techniques have been shown to yield comparable results, one does not have to anticipate substantially different results due to differing techniques [26-28]. Further, LV-EF_echo_ and LV-EF_CMR_ were assessed at different time-points, as patients underwent echocardiography between 24 and 48 hours after the index event, whereas CMR was performed at a median of 72 hours post-reperfusion. Further, assessment of LV-EF using echocardiography is based on geometrical assumptions which lead to less robust results in the presence of regional contractility abnormalities, which is often the case after STEMI. Thus, the difference in LV-EF_echo_ and LV-EF_CMR_ can be most likely explained by the differing techniques and subsequent differing accuracy and slight changes of LV function during the first days after STEMI. In addition, we used CK-MB_max_ to assess enzymatic infarct size, although troponin would have been a good alternative [29-31]. Nevertheless it is unlikely that the inclusion of troponin instead of CK-MB_max_ would have resulted in a significant change in the results as the prognostic value of CK-MB_max_ and its correlation with troponin is well-established [32-34]. Moreover, numerous other parameters assessed by CMR, echocardiography, angiography, laboratory analysis, or functional examinations including right ventricular function, concomitant valve disease, coronary collateralization, N-terminal pro brain natriuretic peptide, or exercise capacity could have been included in the current study. However, from a statistical point of view the analyzable number of parameters should be limited according to sample size and number of events during follow-up. Thus, to conserve an acceptable statistical robustness of the model, we decided to limit our analysis to the best-validated CMR parameters and the most commonly used prognostic markers assessed in clinical routine.

Finally, although we could clearly demonstrate a superior prognostic value of MSI in comparison to other CMR parameters, we cannot elucidate the exact mechanisms linking MSI to adverse clinical outcome, as data on the pathophysiological mechanism behind why MSI is a stronger predictor for adverse clinical outcome than MO or infarct size are scarce. Potential explanations are, for example that individual patient characteristics lead to less MSI and likewise influence adverse clinical outcome such as endothelial function or sensitivity to ischemic pre- and post-conditioning; a higher rate of transmural infarctions and less reduction of infarct size in the months following the index event, in the presence of less MSI, lead to worse functional outcome and substrates for malignant arrhythmias independent of the infarct size itself; or that there is higher local and systemic inflammation in the presence of less MSI. Although theoretically many potential links of MSI with clinical outcome are conceivable, these remain speculative and cannot be fully confirmed by results of basic research. However, despite missing pathophysiological data on the association of MSI and clinical outcome, its prognostic value has been clearly demonstrated in this observational study.

## Conclusions

CMR parameters such as infarct size, MO, MSI, and LV-EF_CMR_, add incremental prognostic value above the assessment of traditional risk markers alone. This allows improved identification of patients at high risk for adverse late outcome and could potentially help to optimize clinical management and subsequent outcome in these patients.

## Abbreviations

CI: Confidence interval; CK-MB_max_: Maximum level of creatine kinase-MB; CMR: Cardiac magnetic resonance imaging; HR: Hazard ratio; IQR: Interquartile range; IR: Inversion-recovery; LV-EF_CMR_: Left-ventricular ejection fraction assessed by cardiac magnetic resonance imaging; LV-EF_echo_: Left-ventricular ejection fraction determined by echocardiography; %LV: Percentage of the left ventricular mass; PCI: Percutaneous coronary intervention; MACE: Major adverse cardiovascular event; MO: Microvascular obstruction; MSI: Myocardial salvage index; STEMI: ST-elevation myocardial infarction; TIMI: Thrombolysis in Myocardial Infarction.

## Competing interests

The authors declare that they have no competing interests.

## Authors’ contributions

SdW: conception and design, data collection and analysis, manuscript writing and final approval of the manuscript. IE: data conception and design, data collection and analysis, manuscript writing and final approval of the manuscript. SD: conception and design, data collection and analysis, manuscript writing and final approval of the manuscript. GF: data collection and analysis, critical revision and final approval of the manuscript. PL: data collection and analysis, critical revision and final approval of the manuscript. TS: data collection and analysis, manuscript writing and final approval of the manuscript. SB: data collection and analysis, manuscript writing and final approval of the manuscript. GS: conception and design, manuscript writing, final approval of manuscript. HT: conception and design, data collection and analysis, manuscript writing and final approval of the manuscript. All authors have read and approved the final form of the manuscript.
